# Supplementary material for: Identification of photoperiod-induced specific miRNAs in the adrenal glands of Sunite sheep (Ovis aries)
Source: Front Vet Sci. 2022 Jul 22;9:888207. doi: 10.3389/fvets.2022.888207 (PMC9354845; doi:10.3389/fvets.2022.888207)
Supplement: Supplementary file 1 [file Data_Sheet_1.ZIP › Supplementary file/miRDeep2 file.zip!/miRDeep2 file/Novel_Structure/SP42A1/Novel_375.pdf]

Provisional ID	:	NC_019459.2_3020
Score total	:	2.4
Score for star read(s)	:	-1.3
Score for read counts	:	0
Score for mfe	:	2.1
Score for randfold	:	1.6
Score for cons. seed	:	
Total read count	:	64
Mature read count	:	58
Loop read count	:	0
Star read count	:	6

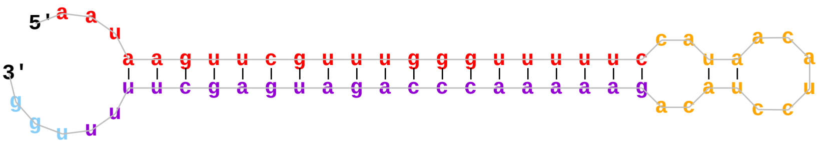

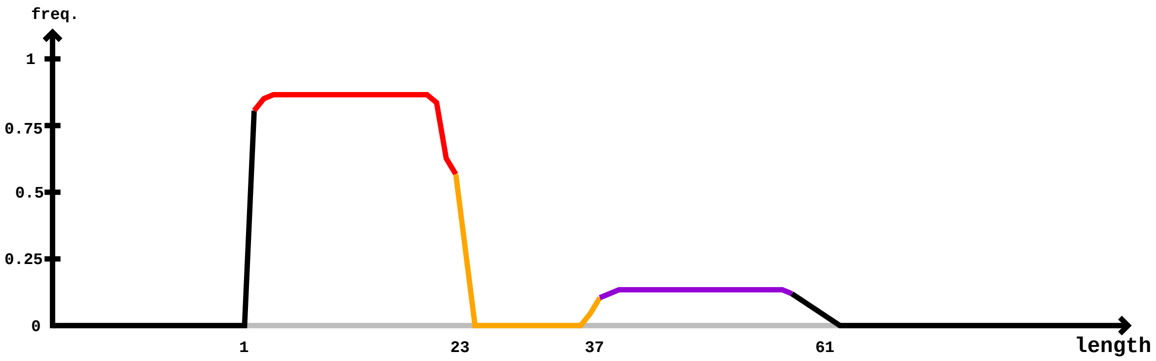


```
                          Mature                          Star
5'- aauacauguuggaguggccaauaaguucguuuggguuuuuccauaacauccuacagaaaaacccagaugagcuuuuuggccaacccaauacuuuauuuccuuuucggguc  -3'  obs
    aauacauguuggaguggccaauaaguucguuuggguuuuuccauaacauccuacagaaaaacccagaugagcuuuuuggccaacccaauacuuuauuuccuuuucggguc       exp
    ......((((((..(((((((.(((((((((((((((((((..((......))..))))))))))))))))))).)))))))..))))))........(((....)))..   reads   mm   sample
    ...................aaAaaguucguuuggguuu........................................................................   2       1    seq
    ...................aauaaguucguuuggguuuu.......................................................................   2       0    seq
    ...................aaAaaguucguuuggguuuu.......................................................................   12      1    seq
    ...................aaAaaguucguuuggguuuuuc.....................................................................   38      1    seq
    ....................auaaguucguuuggguuuuu......................................................................   3       0    seq
    .....................uaaguucguuuggguuuuu......................................................................   1       0    seq
    ......................................................agaaaaacccagaugaAcuuu...................................   1       1    seq
    ......................................................agaaaaacccagaugaAcuuuu..................................   2       1    seq
    .......................................................gaaaaacccagaugaAcuuuu..................................   4       1    seq
    ........................................................aaaaacccagaugaAcuuuuug................................   1       1    seq
    .........................................................aaaacccagaugaAcuuuuugg...............................   1       1    seq
```
